# The Possible Role of IL-17 in Obesity-Associated Cancer

**DOI:** 10.1100/tsw.2010.212

**Published:** 2010-11-16

**Authors:** Tiphaine Gislette, Jiezhong Chen

**Affiliations:** Illawarra Health and Medical Research Institute, School of Health Sciences, University of Wollongong, Australia

**Keywords:** IL-17, obesity-associated cancer, IL-6, Stat3, MAPK, Src/PI3K/Akt

## Abstract

Obesity and overweight have become major medical and social problems. Both are increasing worldwide; two-thirds of the population in developed countries is obese or overweight. Obesity has been associated with many comorbidities, including diabetes and heart disease. Studies have also found that obesity is one of the risk factors involved in increased cancer incidence. Many obesity-related factors are responsible, including increased blood levels of insulin/IGF, IL-6, TNF-alpha, and leptin, and decreased blood levels of adiponectin. Recently, it has been shown that IL-17 levels increase in obese patients. IL-17 is well known to increase carcinogenesis; thus, increased IL-17 levels in obesity may contribute to increased cancer incidence in obesity. IL-17 could activate Src/PI3K, MAPK, Stat3, and PKC, resulting in carcinogenesis. It may also change the microenvironment of tumors. Thus, inhibition of IL-17 may have preventive and therapeutic implications in obese patients.

## INTRODUCTION

Obesity and overweight have become major medical and social problems, so it is necessary to fully understand the mechanisms and comorbidities involved. Obesity is defined as having a body mass index (BMI) more than 30 kg/m$^2$, while overweight is indicated by a 25- to 30-kg/m$^2$ BMI. Levels are increasing worldwide with obesity and overweight together accounting for two-thirds of the population in developed countries[1]. Obesity has been associated with many comorbidities, including hypertension, diabetes, hypercholesterolemia, and heart disease. Studies have also found that obesity is one of the risk factors involved in the increased incidence of many cancers, such as colon cancer, leukemia, and breast cancer[2]. Many factors in obesity have been identified as being responsible for this, including increased blood levels of insulin/IGF, IL-6, TNF-α, and leptin, and decreased blood levels of adiponectin[3]. Recently, it was shown that levels of IL-17 increase in obesity in both humans and mice[4,5].

IL-17 is a well-known proinflammatory cytokine produced mainly by Th17 cells and also by natural killer cells, γδ T, CD4+ and CD8+ T cells[6,7]. On the one hand, IL-17 can promote an immune response and thus plays a key role in the defense against microbial invasion[7,8]. It may also increase immune responses against tumor cells. It has been shown that IL-17 promotes cytotoxic T cells, resulting in tumor regression[9]. IL-17 deficient mice are also more susceptible to developing lung cancer and melanoma, indicating its positive role in the immune response against tumors[10]. On the other hand, IL-17 can increase the growth of tumor cells. Increased IL-17 is associated with chronic inflammation, autoimmune diseases, and cancer[11]. It can promote tumor growth and metastasis[12]. Obesity is linked with chronic inflammatory status, which plays an important role in carcinogenesis. Increased proinflammatory IL-6 in obesity has been demonstrated to play a key role in carcinogenesis[13]; thus, increased blood levels of IL-17 in obesity may also play an important role in obesity-associated cancer[14]. IL-17 and IL-6, as well as their interactions, could be very important among many cancer risk factors associated with obesity. This review summarizes the most recent findings related to IL-17 in obesity, and its possible role in obesity-associated cancer and mechanisms for such roles.

## INCREASED IL-17 IN OBESITY AND POSSIBLE MECHANISMS

Increased blood levels of IL-17 have been reported by several studies. Sumarac-Dumanovic et al. compared the serum cytokines from 26 obese women with those from 20 lean women and found that IL-17 and IL-23 were increased in the obese group, while IL-12 and IFN-γ did not differ[4]. In mice, it was shown that T cells from obese mice induced by a high-fat diet had a greater Th17 T-cell subpopulation and produced more IL-17[5]. Zymosan stimulation also caused more IL-17 production in obese mice, either induced by a high-fat diet or leptin deficiency (ob/ob model) compared to lean mice[15].

Several factors may be responsible for this enhanced production of IL-17 in obesity. First, the secretion of IL-6 by adipocytes and tissue-derived macrophages is increased in obesity[5]. This overexpression of IL-6 increases intracellular levels of Stat3 and retinoic orphan receptor α and γ*t* transcription factors. This, in turn, increases Th17 differentiation[5]. A recent study has also shown that TGF-β–mediated increased IL-6 production promotes naive T-cell differentiation to Th17[16]. Indeed, TGF-β has been demonstrated to play a major role in the balance of Th17 and regulatory T cells[16,17,18]. As Th17 cells produce IL-17, its lineage expansion contributes to enhance IL-17 secretion. Furthermore, it is interesting to note that IL-17 secretion is regulated by a positive feedback mechanism as it increases IL-6 production during adipocyte differentiation[19]. IL-17 can stimulate the production of IL-6 by activation of a few signaling pathways, such as the NF-κB[20], Stat3, or PI3K/Akt pathways[21]. Thus, IL-17 is a cytokine-inducing cytokine, and the interaction between IL-17 and IL-6 could increase the levels of both cytokines[22].

Second, elevated serum levels of amyloid A in obesity also promote the development of the Th17 sublineage via the increased production of IL-23 produced by dendritic cells[5]. IL-23 can act as an inducer of IL-17 or other proinflammatory cytokines (such as IL-22) by activating Stat3 and thus up-regulating the transcription factor retinoic acid–related orphan receptor γt (RORγt), which regulates Th17 cells[23].

Third, TNF-α is increased in obesity and it is closely related to IL-17. In ovarian cancer, TNF-α maintained TNFR1-dependent IL-17 production by CD4+ cells, and increased IL-17 led to myeloid cell recruitment into the tumor microenvironment and enhanced tumor growth[24]. Indeed, in patients with advanced cancer, treatment with the TNF-α–specific antibody infliximab substantially reduced plasma IL-17 levels. Thus, IL-17 could be increased by TNF-α in obesity. Further experiments are required to confirm the role of increased TNF-α on IL-17 levels in obesity.

## THE EFFECT OF INCREASED IL-17 IN OBESITY-ASSOCIATED CANCER

Evidence has shown that increased IL-17 plays an important role in increased cancer incidence and thus its increase in obesity may promote obesity-associated cancers[4,14,25]. Increased blood levels of IL-17 have also been observed in the presence of a human colonic bacterium called *Bacteroides fragilis* (BF). The toxin secreted by the bacteria is responsible for the induction of IL-17 via the activation of the Stat3 and NF-κB pathways[26]. The increase in IL-17 was demonstrated to play a key role in BF-induced colon cancer. A recent study also showed that IL-17 plays an important role in the development of multiple myeloma[27]. In melanoma cell culture, the addition of IL-17 promoted cell growth and was also shown to increase colony formation. In a murine xenograft model, injection of IL-17 also increased the growth of human multiple myelomas[27]. Furthermore, in lung cancer, the expression of IL-17 is associated with a poor prognosis, and this may be caused by increased angiogenesis and lymphangiogenesis[28].

Increased blood levels of IL-17 in obesity may increase cancer incidence via the activation of various signaling pathways[14]. It has been demonstrated in other cases that IL-17 can activate the Src/PI3K/Akt/NF-κB, MAPK, Stat3, and COX-2 pathways. These pathways are well known to play an important role in the carcinogenesis of many cancers[29,30]. However, how increased IL-17 in obesity contributes to activate these pathways and is further associated with carcinogensis has not been well studied.

## Src/PI3K/Akt/NF-κB Pathway

IL-17 can activate Src to increase carcinogenesis[14]. Src activation modifies both cell growth and the cytoskeleton[31,32]. One major Src-activating pathway is PI3K/Akt. Src can phosphorylate PI3K to activate the PI3K/Akt signaling pathway and induce IκB phosphorylation. Since IκB undergoes ubiquitin-dependant degradation, it releases NF-κB that can translocate to the nucleus and regulate genes that encode proinflammatory mediators[33]. These can increase cell resistance to apoptosis and thus lead to carcinogenesis. NF-κB also seems to play an important role in cytokine-induced gene expression. In fact, IL-17 activates NF-κB in chondrocytes, leading to joint inflammation, and this is associated with decreased levels of IκB[34].

Moreover, the PI3K/Akt signaling pathway activates the oncogenic protein Mdm2 to block p53. Due to the tumor-suppressing properties of p53, decreased levels of this protein will lower apoptosis[3]. In patient samples, it has been shown that levels of serum IL-17 are inversely related to p53[35]. The activated PI3K/Akt pathway also blocks the Fas-associated death domain protein that inhibits the cellular apoptosis pathway[3]. The PI3K/Akt pathway is responsible for mTOR activation, which in turn leads to increased protein translation and GSK3β phosphorylation to increase the cell cycle[3,36].

In HPV-associated lung cancer, IL-17 is increased by the HPV E6 protein[37]. Increased levels of IL-17 promote carcinogenesis of non–small cell lung cancers via the PI3K/Akt/Mcl1 pathway[37]. Thus, it is possible that increased IL-17 in obesity may contribute to obesity-associated cancer via this pathway.

## MAPK Pathway

IL-17 also induces MAPK activation, which is known to play an important role in the carcinogenesis of various cancers[14,38]. The MAPK family is mainly composed of three cascades, including p38 MAPK, ERK, and JNK[39]. The pathway is known to mediate intracellular signal transduction in response to various extracellular stimuli, such as growth factors, environmental stress factors, cytokines, hormones, and oxidants[40]. To be activated, the MAPK pathway requires a dual phosphorylation on threonine and tyrosine residues by MAPKK and the MAPKK is, in turn, activated by phosphorylation on serine and threonine residues by MAPKKK[41]. Once activated, they translocate to the nucleus to modulate the activity of nuclear transcription factors and kinases, leading to changes in cell functions, e.g., cytokine expression, proliferation, and apoptosis[39].

Evidence has shown that IL-17 can activate MAPK. Binding of IL-17 to human renal epithelial cells (hRECs) is responsible for phosphorylation of Src kinases[42]. In turn, these activated Src kinases may phosphorylate the SHC-GRB2-SOS complex that contributes to Ras/MAPK activation. It leads to increased expression and production of proinflammatory cytokines in hRECs, which include IL-6, IL-8, and MCP-1 to influence cell proliferation[42].

A recent study has shown that IL-17 causes an enhanced phosphorylation of the three MAPK pathway components (p38 MAPK, ERK1/2, and JNK) to increase secretion of proinflammatory chemokines MCP-1 and MIP2 in mesangial cells[43]. The important role of the MAPK pathway is demonstrated in that inhibitors of p38 MAPK and ERK1/2 abolish such an effect. These chemokines are associated with chronic inflammatory diseases and cancer; thus, IL-17 could activate MAPK and its downstream products to increase inflammatory processes[44,45].

### Stat3 Pathway

The third pathway activated by IL-17 is Stat3. Increased levels of IL-17 stimulate cancer formation via the Stat3 pathway[33]. The IL-17 receptor activates JAK family kinases that are followed by Stat3 protein phosphorylation[46,47,48,49]. It has also been shown that IL-17–activated IL-6 in turn activates Stat3[50]. Studies have demonstrated that Stat3 persistent activation in cancer leads to tumor-promoting inflammation[33,51]. Its activation generates up-regulation of key genes involved in cell proliferation and survival, and overexpression of antiapoptotic proteins like Bcl-xL and cycle regulators such as Myc[52]. In addition, activation of Stat3 also leads to antitumor immunity suppression[33]. It has been demonstrated that ablated Stat3 decreased the IL-17–induced proinflammatory effect in mice[53].

### Protein Kinase C Pathway

A member of the IL-17 cytokine family, IL-17F, has an effect on pulmonary microvascular endothelial cells (PMVECs) via the signaling pathways previously described (MAPK and protein tyrosine kinase) and also via another pathway: the protein kinase C (PKC) pathway[54]. Stimulation of PMVECs by IL-17F activates PKC. PKC activation leads to an up-regulation of mRNA and protein expression of Src-suppressed C kinase substrate (SSeCKS) that is translocated from the cytoplasm to the membrane. This translocation, in addition to its increase during the cell cycle to reorganize the actin skeleton[55], contributes to endothelial hyperpermeability[54]. Other studies have revealed that, more generally, IL-17F induced cytokine production from endothelial cells, such as IL-6, IL-8, or granulocyte colony-stimulating factor[56], and regulated angiogenesis[57]. Angiogenesis is an important process[58] and, thus, it is interesting to investigate how increased IL-17 in obesity increases angiogenesis via the PKC pathway.

## TUMOR MICROENVIRONMENT

The tumor microenvironment influences tumor growth and metastasis. IL-17 can promote tumor development through changes in the tumor microenvironment[59]; increasing tumor growth by inducing angiogenesis[60,61]. However, a recent study has controversially shown that an IL-17–changed tumor microenvironment delayed the growth of pancreatic cancer[62]. Mice injected with IL-6–transduced pancreatic cancer cells had a longer overall medium survival rate compared with those injected with wild-type pancreatic cells, probably because IL-6 produced more Th17 than T-regulatory cells, thus increasing antitumor immunity. Studies in 21 ovarian cancer patients also showed that increased IL-17 cells are correlated with increased effector T cells, as well as increased CXCL9, CXCL10, and decreased T-regulatory cells[63]. These changes provide anticancer immunity. The dual effect of IL-17 on the tumor microenvironment may depend on different conditions[64]. It is not yet known how increased IL-17 changes an obesity-associated microenvironment.

## THERAPEUTIC IMPLICATION

The importance of IL-17 in obesity-associated cancer indicates that it may be manipulated for prevention and treatment. In mice with APC min/+, ablation of IL-17 reduced tumor development with decreased proinflammatory cytokine[65]. It also corrects immune defects in these mice. Another study showed that B16 melanoma and MB49 bladder carcinoma growth was reduced in IL-17–/– mice, indicating the preventive effect of IL-17 inhibition[50]. It will be interesting to investigate the effect of IL-17 inhibition on obesity-associated cancer. Attention should also be given to the changes in the immune system and adipocyte differentiation.

Recent studies have revealed that IL-17 may exert an inhibitory effect on adipocyte differentiation[19]. Indeed, increased secretion of IL-17 causes an up-regulation of COX-2, an enzyme catalyzing the conversion of arachidonic acid into PGE2. PGE2, in turn, inhibits adipocyte differentiation. In addition, increased blood levels of IL-17 stimulate lipolysis of adipocytes, thus playing a role in modulating the metabolic state of differentiated adipocytes to IL-17[19].

## CONCLUSION

Obesity is associated with multiple cancer risk factors. IL-17 could be a new addition to this list. Increased IL-17 in obesity could be caused by increased IL-6, amyloid, and TNF-α. It may activate several signaling pathways to increase cancer incidence in obesity, including PI3K/Akt, MAPK, Stat3, and COX-2. Thus, inhibition of these pathways may have preventive and therapeutic implications in obesity-associated cancer.
